# Comparative measurement of CNP and NT-proCNP in human blood samples: a methodological evaluation

**DOI:** 10.1186/1477-5751-12-7

**Published:** 2013-04-01

**Authors:** Andreas Kuehnl, Jaroslav Pelisek, Martin Bruckmeier, Wajima Safi, Hans-Henning Eckstein

**Affiliations:** 1 Clinic for Vascular and Endovascular Surgery, Klinikum rechts der Isar, Technische Universität München, Ismaninger Strasse 22, Munich, 81675, Germany

**Keywords:** CNP, NT-proCNP, Sample processing

## Abstract

**Background:**

C-type natriuretic peptide (CNP) has anti-inflammatory, anti-proliferative, and anti-migratory properties. During the past years, CNP has attained an increasing interest by many research groups, especially in the cardiovascular field. Nevertheless, still no reliable data exist on the difference of CNP concentration between serum and plasma samples. Also, the influence of delayed blood sample proceeding is unknown. The aim of this study was to investigate the difference of CNP and NT-proCNP concentrations between serum and plasma samples. In order to identify potential methodological bias, this study should also validate the stability of CNP and NT-proCNP in full blood samples stored at room temperature.

**Findings:**

Triplets (serum, plasma, full blood) of fasting blood samples from 12 healthy male individuals were collected. Analysis of CNP and NT-proCNP concentration was performed immediately following sampling, and after 30 minutes or 2 hours of storage at room temperature. Mean serum concentrations at baseline were 0.997 ± 0.379 ng/ml for CNP and 58.5 ± 28.3 pg/ml for NT-proCNP. Furthermore, NT-proCNP concentration did not change significantly during the allotted time and did not differ between serum, plasma, and full blood samples. At baseline, concentrations of CNP were significantly different between samples containing either sodium-citrate or EDTA as a clotting inhibitor (1.933 ± 0.699 ng/ml vs. 0.991 ± 0.489 ng/ml, p = 0.001).

**Conclusions:**

CNP and NT-proCNP are stable for at least two hours, even when sample processing is delayed or blood probes are stored at room temperature. NT-proCNP assay demonstrated more consistent and reliable data and should therefore be preferred for usage in clinical applications. Nevertheless, as recommended for ANP and BNP, immunoassays for CNP should also be standardized or harmonized in the future.

## Findings

### Introduction

C-type natriuretic peptide (CNP), identified in 1990 by Sudoh et al., is the most ancient member of the natriuretic peptide family [1]. CNP selectively stimulates the human natriuretic peptide receptor 2 (NPR2), which activates the cGMP-dependent kinase (cGK) by elevating intracellular concentration of cGMP [2]. In addition, CNP binds NPR3 and in turn deactivates protein kinase A by G_i_-protein dependent inhibition of adenylate cyclase [3]. CNP has anti-proliferative and anti-migratory properties [4] and was also identified as an endothelium derived relaxing factor [5]. Furthermore, CNP inhibits neointimal restenosis, reduces vascular constrictive remodeling and cardiac ischemia-reperfusion injury [6,7]. CNP plays an important role in bone growth, reproduction, nerve growth, re-endothelialisation, as well as renal, pancreatic, and cardiac function [2,3]. In addition, CNP has recently been recognised to be involved in the development of atherosclerotic plaque formation [8,9].

Due to the plethora of CNP functions, this peptide has attained increasing interest in cardiovascular research during the past years. However, studies concerning CNP concentrations in blood samples are inconsistent. Some of these studies measured concentrations of CNP or its amino-terminal pro-peptide (NT-proCNP) in serum samples and others used plasma samples [10-22]. Unfortunately, details on potential delays during blood sampling and subsequent sample processing have not yet been reported. In the available literature thus far, no data exist on the difference of CNP/NT-proCNP concentrations between serum and plasma samples. Also, the influence of the delay of blood sample processing remains unclear. In addition, CNP concentration in serum and plasma samples varied markedly (Table 1). Therefore, the aim of this study was to answer the following methodological questions: (1) Does delayed processing of the blood samples stored at room temperature cause bias? (2) Is there any difference between serum and plasma concentrations of CNP or NT-proCNP? (3) Are these differences time dependent?

**Table 1:** Overview of the reported baseline blood concentrations of CNP and NT-proCNP (mean ± SD)

**Author, year**	**Age**	**n**	**Subgroup**	**CNP (pg/ml)**		**NT-proCNP (pg/ml)**	
				**Serum**	**Plasma**	**Serum**	**Plasma**
Del Ry, 2012 [11]	0-3d	41	newborns		11.6 ± 2.1*^R^		
	4-30d	24	newborns		16.4 ± 3.7*^R^		
	1-12 m	22	infants		15.4 ± 2.7*^R^		
	1-12	32	childrens		13.6 ± 2.3*^R^		
	64 ± 1	32	healthy adults		7.4 ± 1.0*^R^		
Del Ry, 2011 [10]	64 ± 1	130	heart failure		7.86 ± 0.41*^R^		67.1 ± 7.36
	63 ± 2	19	diabetes		8.70 ± 1.62*^R^		51.5 ± 5.75
	52 ± 1	24	liver cirrhosis		4.97 ± 0.55*^R^		78.4 ± 19.9
	61 ± 2	73	healthy		2.35 ± 0.11*^R^		45.5 ± 1.84
Olewicz-Gawlik, 2010 [17]	60 ± 15	40	RA			105 ± 189	
		30	healthy			50 ± 35	
Vlachopoulos, 2009 [21]	57 ± 10	52	erectile dysfunction				2.28 ± 0.87
	matched	31	healthy				3.70 ± 0.76
Palmer, 2009 [19]	64 ± 9	120	cardiovascular disease		1.43*^R,a^		187.0^a^
Dietmann, 2008 [14]	0.8-3.6	50	Malaria, severe				120 ± 72
	0.8-2.0	39	Malaria, uncomplicated healthy				125.0 ± 49
		25					
	0.8-6.4						
							155 ± 70
Yagci, 2008 [22]	34 ± 6	26	healthy	800 ± 270^E^			
	34 ± 9	28	Behcet, active	490 ± 120^E^			
		25	Behcet, inactive	650 ± 200^E^			
Del Ry, 2007 [12]	62 ± 2	51	ILVD		7.0 ± 0.9*^R^		
	61 ± 2	60	healthy		2.5 ± 0.12*^R^		
Olney, 2007 [18]	13 ± 2	10	GHD baseline				339 ± 40
			GHD under therapy				622 ± 157
	14 ± 1	11	healthy				432 ± 64
Lupattelli, 2006 [16]	35-60	44	Hyperlipidemia		4100 ± 5800*^E^		
	matched	20	healthy controls		5700 ± 3300*^E^		
Del Ry, 2005 [13]	61 ± 2	21	healthy		2.7 ± 0.2*^R^		
Prickett, 2001 [20]	33 - 91	22	heart failure				105 ± 5.4
	25 - 55	16	healthy				80 ± 3.2
Gülberg, 2000 [15]	57 ± 2	20	liver cirrhosis, RI		2.7 ± 0.4*^R^		
	52 ± 2	20	liver cirrhosis, no RI		3.0 ± 0.2*^R^		
	45 ± 6	11	healthy		4.2 ± 0.4*^R^		
present study	29 ± 2	12	healthy	997 ± 379^E^	991 ± 489*^E^	58.5 ± 28.3	60.3 ± 23.9
					1933 ± 699°^E^		

At baseline, CNP concentrations are measured in blood samples anticoagulated either with EDTA(*) or sodium-citrate (°). Concentration of CNP was measured either with RIA-assay (R) or ELISA-assay (E). Unless otherwise indicated (*d* = days, m = months), age is given in years. a = arterial blood sample, *CVRF* = cardiovascular risk factors, *RA* = rheumatoid arthritis, *ILVD* = idiopathic left ventricle dysfunction, *RI* = renal insufficiency, *GHD* = growth hormone deficiency.

### Methods

We studied 12 male volunteers (mean age 29 ± 2 years, normal weight, no drug therapy, no history of cardiovascular or other diseases, 3 smokers). From all investigated subjects informed consent was obtained. Triplets of fasting blood samples were taken from all volunteers at 8 o’clock in the morning. Blood samples were collected using the 7.5 ml S-Monovette® collection system (Sarstedt, Nümbrecht, Germany) containing either clotting activator (Kaolin) for serum samples, sodium-citrate for plasma samples or Potassium–EDTA for full blood samples. All samples for baseline analyses were immediately centrifuged at 2,000 × g for 10 min at 4°C and supernatant was stored at −70°C until analysis. For mid-term analyses (30 minutes or 2 hours), plasma samples were centrifuged at 2,000 × g for 10 min at room temperature. Supernatant was removed and stored at room temperature for 30 min or 2 hours. Following blood clotting, serum samples were processed identically to plasma samples. Supernatant was saved and also stored at room temperature for 30 min or 2 hours. Full blood samples were stored at room temperature without centrifugation. After 30 min or 2 hours, full blood samples were centrifuged at 2,000 × g for 10 min at 4°C. Supernatant was removed and stored at −70°C until analysis. The concentration of CNP was measured using the CNP-22 EIA Kit (#EK-012-03, Phoenix Pharmaceuticals, Karlsruhe, Germany) according to the manufacturer’s protocol. NT-proCNP concentration was measured using the NT-proCNP EIA Kit (Biomedica, Vienna, Austria) according to the manufacturer’s protocol. One-way ANOVA was used for comparison between groups. For pairwise comparisons, the *t*-test was applied. Data are shown as means ± standard deviation (SD) or 95% confidence interval (95%-CI). Statistical significance was assumed at p < 0.05. Data were analyzed using MedCalc® for Windows, Version 10.0.1.0 (MedCalc Software, Mariakerke, Belgium).

### Results

**Figure 1:**
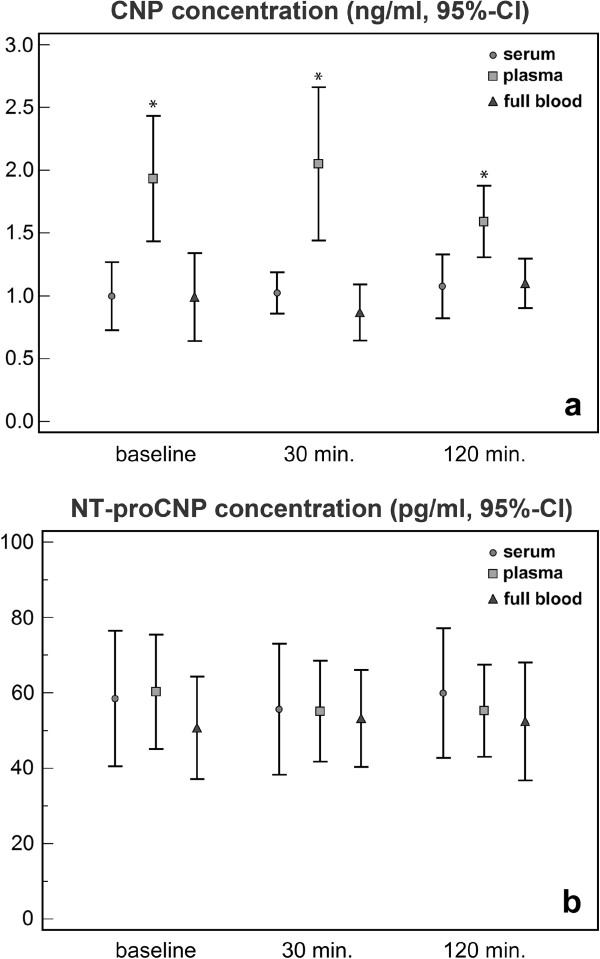
**Concentrations of CNP and NT-proCNP in blood samples. a**) Concentration of CNP in serum, plasma and full blood samples (means, 95% confidence intervals). CNP concentrations in plasma samples are significantly higher compared to serum and full blood samples at all time points (ANOVA, p = 0.001 at baseline, p < 0.001 at 30’ and p = 0.003 at 120’). There is no significant difference between serum and full blood samples at any time point (ANOVA, p > 0.05). **b**) Concentration of NT-proCNP in serum, plasma and full blood samples (means, 95% confidence intervals). There is no significant difference between the groups at any time point (ANOVA, p > 0.05).

As shown in Figure 1a, baseline concentrations of CNP in serum, plasma, and full blood samples were 0.997 ± 0.379 ng/ml, 1.933 ± 0.699 ng/ml, and 0.991 ± 0.489 ng/ml, respectively. Plasma concentrations of CNP were significantly higher compared to serum and full blood samples at all time points (ANOVA, p = 0.001 at baseline, p < 0.001 at 30’ min. and p = 0.003 at 120’ min.). No significant difference was observed between serum and full blood samples at any time point (ANOVA, p > 0.05). Baseline concentrations of NT-proCNP in serum, plasma, and full blood samples were 58.5 ± 28.3 pg/ml, 60.3 ± 23.9 pg/ml, and 50.7 ± 21.4 pg/ml, respectively (Figure 1b). No significant difference was found between the groups at any time point (ANOVA, p > 0.05). In full blood samples, concentration of lactate increased significantly after 2 hours compared to baseline value (3.2 ± 0.8 mM vs. 2.0 ± 0.6 mM, p < 0.001). In addition, pH-value decreased from 7.34 ± 0.03 at baseline to 7.29 ± 0.03 after 2 hours (p < 0.001).

### Discussion

Our study revealed that CNP and NT-proCNP are stable in serum and in full blood samples for at least two hours at room temperature. Consequently, the stability of the CNP peptide is most likely not affected by any delay in sample processing for at least two hours. ProCNP has already been reported (manufacturer’s protocol, unknown storage conditions of blood samples) to be stable for at least 2.5 hours. Accordingly, our experiments confirmed the stability of NT-proCNP even at room temperature. The concentration of NT-proCNP in all sample types remained constant during the time course for up to 2 hours, indicating long lasting stability of this protein. As listed in Table 1, the mean concentration of NT-proCNP (56.5 ± 24.3 pg/ml) measured in this study was within the reported range (see Table 1) of healthy individuals (3.7-432 pg/ml).

In contrast to NT-proCNP, the concentration of CNP was significantly higher in plasma samples compared to serum and full blood samples (Figure 1). So far, neither our group nor manufacturers technical service found any evidence concerning the influence of sample type on the ELISA assay used in this study. However, at baseline, supernatant of plasma and full blood samples were identical with the exception of type of blood clotting inhibitor used. This inhibitor was sodium citrate in the plasma group and EDTA in the full blood sample group. Full blood samples revealed comparable results to serum samples (p = 0.98). Therefore, it is most likely that sodium citrate may significantly influence the measured concentration of CNP in plasma and consequently falsify the results achieved by this technique.

Unexpectedly, baseline concentrations of CNP in both plasma and serum samples were about a thousand-fold higher than in other reports (Table 1) [10-13,15,19]. In all of these studies the Radioimmunoassay (RIA-Kit) was used for the determination of CNP concentrations. In contrast, in two other studies the measured CNP concentrations in serum and plasma samples were comparable to our results with respect to the magnitude of dimension [16,22]. Interestingly, in the latter studies, the same ELISA-Kit was used as in our investigation. Even after extensive evaluation of various internal and external factors, no satisfied explanation of this discrepancy could be found. Control measurements using exogenous CNP in human serum revealed a non-significant measurement error of +7.8% (95%-KI: [−1.9%]–[+17.5%]). The CNP peptide used for the reference curve was synthesized by the manufacturer itself (Phoenix). In contrast, the CNP peptide used as the “exogenous” control was provided by Bachem. As assured by both manufacturers, sequences of amino acids were identical and disulfide bonds were formed in both peptides.

However, results of internal control measurements can be summarised as follows: First, the control measurements performed in our study revealed that the ELISA kits were used properly, in accordance with the manufacturer’s instructions. Second, measurements using control samples confirmed suitability of standard curve applied. Third, control serum samples containing exogenous CNP revealed acceptable accuracy and results were within the expected order of magnitude. Fourth, a higher concentration of CNP in plasma samples is most likely an artefact caused by sodium-citrate.

As reported by Clerico and co-workers, comparably great differences in results among different RIA and EIA methods are also known for, e.g. ANP and BNP [23-26]. Clerico and co-workers concluded that the large differences in these results are most likely due to the specificity of monoclonal or polyclonal antibodies used, the design of immunoassay system (competitive vs. non-competitive assay), the analytical matrix (serum vs. EDTA vs. heparinized plasma) used, and the plethora of circulating forms of natriuretic peptides, as previously reported for ANP and BNP immunoassays [23,26,27]. These methodological sources of bias may also be conceivable for CNP and NT-proCNP assays and thus explain the great difference in results in the cited studies (Table 1).

#### Limitations of the study

We acknowledge that a sample size of 12 is too small to conclude that there is no difference at all. However, from a statistical point of view, it would be important to specify how large the difference must be to be of medical relevance. To our knowledge, the latter is not yet clearly defined. Therefore, the means and 95%-confidence intervals of all measures were given in the figure to enable every reader to draw his own interpretation from the data as well.

In reference to serum and plasma concentrations, it must be mentioned that the values reported in Table 1 were measured with different methods (RIA, ELISA) in patients suffering from different diseases. It would have been very helpful to compare CNP-RIA with CNP-ELISA assays directly using the same specimens, because a thousand-fold difference in the order of magnitude remains noticeable. Unfortunately, RIA assays measurements were not available in our laboratory. For NT-proCNP concentration, the latter comparison was investigated by Olney and co-workers[28] showing that the commercial ELISA (Biomedica Medizinprodukte GmbH & Co KG, Vienna, Austria) gave values that were an average of 21% (range 11-52%) of the RIA values. Cross-validation of the reference standard of the ELISA kit using RIA assay showed a disagreement of 15% [28].

#### Summary

Measurements of CNP and NT-proCNP were most likely unaffected by delay of sample procession or type of blood sample (except for CNP in plasma samples). For plasma samples, only EDTA anticoagulated blood samples were suitable for the CNP ELISA assay used in this study. Consequently, the concentration of CNP and NT-proCNP could be used in clinical applications in order to determine CNP effects. It should also be considered that the concentration of NT-proCNP, being only a by-product of the active peptide, may conceal the true nature of CNP. However, discrepancy in measured CNP concentrations determined either by RIA-assays or by ELISA-assays still remains unexplained but seems most likely to be due to methodological issues. Therefore, as recommended for ANP and BNP [23], immunoassays for CNP need also to be standardized or harmonized in the future.

## Competing interests

All authors declare that they have no competing interests.

## Authors’ contributions

AK: Study design, acquisition of data, data analysis/interpretation, drafting the manuscript. JP: Study design, acquisition of data, critical revision of the draft. MB: acquisition of data, drafting the manuscript. WS: acquisition of data, drafting the manuscript. HHE: Study design, data interpretation, critical revision of the draft. ALL: Final approval of the manuscript.
